# First Record of the Genus *Zygina* from a Neotropical Region on *Populus spp*.: Taxonomic and Biological Characteristics

**DOI:** 10.1673/031.011.8801

**Published:** 2011-07-11

**Authors:** M.I. Catalano, M.E. Brentassi, S. L. Paradell, A.M.M. Remes de Lenicov

**Affiliations:** Universidad Nacional de La Plata. Facultad de Ciencias Naturales y Museo. División Entomología Paseo del Bosque s/n. La Plata - Buenos Aires. Argentina

**Keywords:** Argentina, Cicadellidae, chlorosis, polymorphism, *Populus alba*, *Populus nigra*, Typhlocybinae, *Zygina nivea*

## Abstract

The typhlocybine, *Zygina nivea* Mulsant & Rey 1855, was found in urban areas of Argentina colonizing trees of poplar (*Populus alba* L. and *P. nigra* L.). This is the first mention of the genus *Zygina* Fieber from the Neotropical region. In this paper redescription of the male, description of the female, distributional and host plant data, and behavioural observations of this species are given.

## Introduction

The cosmopolitan subfamily, Typhlocybinae, is a diverse group composed of mostly tiny, delicate leafhoppers comprising about 5000 described species (Oman et al. 1990). The largest tribe of Typhlocybinae is the Erythroneurini, with 164 genera and nearly 2000 described species (Dietrich & Dmitriev 2006). The previous documented Argentine fauna of Erythroneurini is so far comprised of one genus and three species (Dietrich & Dmitriev 2007). Two Palearctic species are cited to New World, *Zygina flammigera,* from Washington and British Columbia States (Hamilton 1983) and *Alnetoidia alneti* from Nova Scotia (Dmitriev & Dietrich 2009)

Young (1952) includes in the *Zygina* genus all those leafhoppers with a single broad extension of style apex, most recently Dworakowska (1970) narrowed the genus to include only Palearctic species with the following combination of characters: male pygofer with conspicuous setae laterally and on dorsal membrane; dorsal appendage small, slender, and not movably articulated; subgenital plate without basolateral projection and macrosetae distributed in midlength; style apex with two points; and preapical lobe weakly developed including only Palearctic species (Dworakowska 1970; Dietrich & Dmitriev 2006). All those species were transferred to several newly described genera by Dietrich & Dmitriev, 2006.

Typhlocybinae feed preferentially on the contents of leaf parenchyma cells of their host plants; causing stipling injury to numerous crops when the populations reach high densities, while other species cause a more severe injury known as hopperburn (Backus et al. 2005). In Argentina, the first studies on the feeding behavior of a typhlocybine were carried out for *Typhlocybella maidica* on maize (Brentassi et al. 2010). This species feeds primarily from the mesophyll cell using the cell rupturing feeding strategy causing stipling in corn. Moreover, several typhlocybine species are vectors of phytoplasms and viruses (Nielson 1979; Arocha et al. 2005; Weintraub & Beanland 2006; Alhudaib et al. 2009). Due to the severity of the feeding injury, species of *Zygina* are used in Australia for biological control agents for the “bridal creeper” weed, *Asparagus asparagoides* (L.) W. Wight (Asparagaceae) (Witt & Edwards 2000; Kleinjan et al. 2004).

The purpose of this paper is to redescribe the male of *Zygina nivea,* to describe the unknown female, and to provide new distributional and host plant data. Also, field and laboratory observations on the behaviour and the population density of this species on poplar plants are given.

## Materials and Methods

The specimens examined were collected from locations in the Buenos Aires province (Capital Federal and La Plata) with manual aspirators in plants of *Populus alba* and *P. nigra* in summer and autumn of 2009 and 2010.

A series of female and male specimens of *Z*. *nivea,* most of them found in copula on the host plant, were considered as “reference specimens” and used for the descriptions and illustrations.

For the identification, the abdomen was removed from individual specimens and cleared with ten percent caustic potash, using methods similar to those described by Young (1952). Cleared material to be studied was placed in glycerin on a depression slide. The illustrations were drawn using a light microscope with a camera lucida. Measurements were taken from ten specimens of each sex and were expressed in millimetres; some measurements, for example: valve length, were interpreted as ratios. Body length was measured from the anterior margin of the crown to the tip of the forewing in repose. The terminology of Young (1952) and Southern (1982) were used to describe the main morphological characters of the male genitalia; and that of Balduf (1934) of the ovipositor. The series of described specimens is deposited in the collection of Museo de Ciencias Naturales de La Plata (MLP).

The adults were raised in Petri dishes, having access to poplar leaves under controlled conditions (24 ± 2° C, 50–60% RH; 16:8 L:D photoperiod) in the Entomology Division at Museo de Ciencias Naturales de La Plata.

Due to adults having different body coloration, and in order to determine patterns of variation during development, 20 fifth-instars nymphs were selected and placed individually in Petri disks with a leaf of poplar. To estimate coloration changes, the insects were observed and photographed at intervals of 24 hours after adult emergence using a stereoscopic microscope.

The density of *Zygina nivea* on *P. alba* and *P. nigra* was estimated by counting the number of insects/leaf on ten leaves of each of ten plants of each poplar species.

## Results

**Figure 1.**
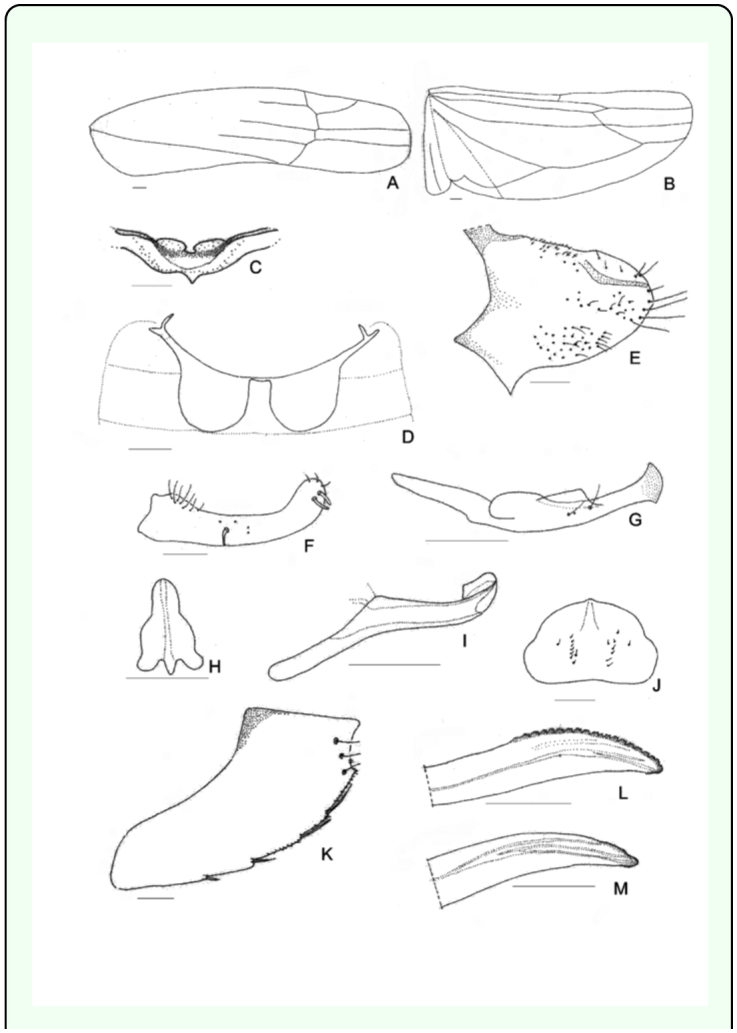
*Zygina nivea,* (a) forewing, (b) hind wing. Male: (c) First sternal complex, 1S; (d) Second sternal apodemes, 2S; (e) pygofer, lateral view; (f) subgenital plate; (g) style; (h) connective; (i) aedeagus. Female: (j) sternite VII; (k) pygofer, lateral view; (l) large valve; (m) small valve. (Figures a, b: 10X; c, d, e, f, j, k: 20X; g, h, i, l, m: 40X) Scale bar = 0, 1 mm. High quality figures are available online

**Figure 2.**
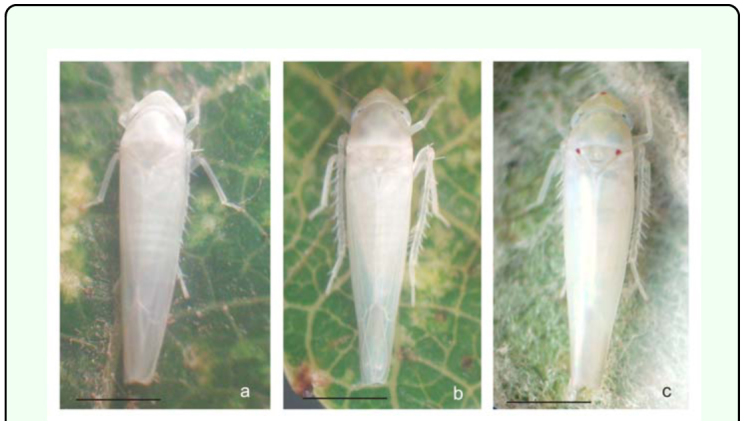
Male of *Zygina nivea,* (a) 0–1 day old, (b) 2–3 day old, (c) 4–5 day old. Scale bar = 1 mm High quality figures are available online

**Figure 3.**
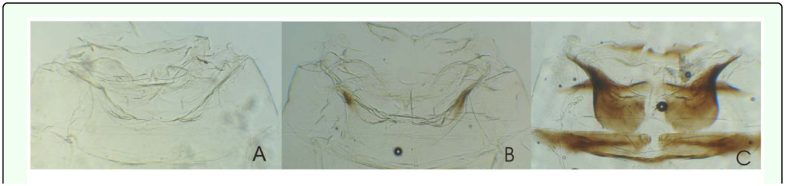
*Zygina nivea,* sternal apodemas: (a) 0–1 day old, (b) 2–3 day old, (c) 4–5 day old. High quality figures are available online

**Figure 4.**
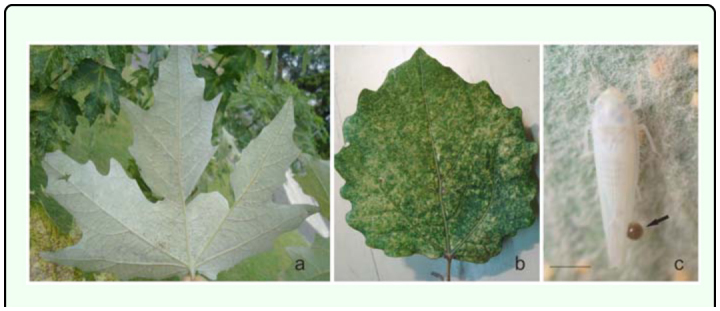
(a) Poplar leaf with nymphs and adults of *Zygina nivea*; (b) feeding damage; (c) excretion. Scale bar = 1 mm.

**Genus: *Zygina* Fieber, 1866**
Type species: *Typhlocyba nivea* Mulsant & Rey, 1855; by monotypy
***Zygina nivea*** (Mulsant & Rey, 1855)
*Typhlocyba nivea* Mulsant & Rey, 1855
Synonymy: *Typhlocyba punctulatum* Mulsant & Rey, 1855; *Zygina punctulum cruoris* Rey, 1891; *Erythroneura nivea dorsuaria* Ribaut, 1936; *E. nivea mycthemera* Ribaut, 1936
**Male**
  Measurements: Male's total length: 3.4 – 3.5 mm. Coloration: Green yellowish with a red spot in the apex of the crown, and two red spots on each side at base of the scutellum. Crown slightly produced medially. Forewing (Figure la): inner apical cell with base oblique, more proximal than base of second apical cell; second apical cell narrower than either adjoining apical cell; third apical cell broader at apex than base; outer apical cell short and small, not attaining wing apex. Hindwing (Figure lb): posterior branch of R fused with vein M_1+2_; vein Cu_2_ confluent with submarginal vein in basal half of wing, vannal veins fused; submarginal vein absent at wing apex, confluent with apex of vein Cu_1_ apically.
  First sternal complex (1S) (Figure 1c) with sternal bar lightly sclerotized with apical third curved upward, dorsal apodeme rounded and well development and more sclerotized in the middle with broad medial notch not reaching sternal bar. Second sternal apodemes (2S) (Figure 1d) short, reaching segment 3, medial margin convex; medial notch broad; tips rounded.
  Male genitalia: Pygofer (Figure 1e), in lateral view, rounded caudal margin with shaped hair-like setae on the posterior margin and several microsetae on disk; a small, slender and not movably articulated, dorsal appendage not exceeding the caudal margin. Subgenital plates (Figure 1f) narrow, with one macrosetae near the apical half of its length and two others near the tips; some irregularly dispersed short microsetae between them and a row of five to seven thin, long microsetae on the outer margin at the base. Style (Figure 1g) with preapical lobe distinct, with two or three microsetae at the base, and single flat truncate apical extension. Connective (Figure 1h) triangular with two lateral lobes in the base. Aedeagus (Figure 1i) short without processes; dorso-apical margin expanded and recurved anteriorly, oval gonopore subterminal.
**Female**
  Measurements: Female's total length: 3.5 – 3.6 mm. Coloration and shape of the body very similar to male.
  Female genitalia. Seventh sternum (Figure 1j) conically produced. Pygofer (Figure 1k), in lateral view, with 3 or 4 macrosetae in row on ventro margin and 2 or 3 setae on caudal margin. Medium valve large 12 times longer than broad, curved in apical part, rounded at apex; large valve (Figure 11) with 18 robust and regular teeth, with two denticles on each one, ventral margin with several small teeth, with one longitudinal sclerotized bar ramified in the apex; small valve (Figure 1m) with numerous, small and countless teeth in dorsal margin and one sclerotized bar.
**Material examined:** ARGENTFNA. Buenos Aires: Capital Federal, 4/18/2009, Brentassi-Catalano col., 10 males, 10 females. ARGENTINA. Buenos Aires: La Plata, 3/10/2010, Brentassi col., 5 males, 5 females.
**Polymorphism**
**Changes in coloration** (Figure 2): Immediately following adult emergence, body and forewing coloration is whitish; the coloration changes to yellow-greenish in insects between 1 and 3 days old; at 4 days old, the red spots on crown and scutellum begin to appear, and the thoracic sternites and urotergites change from whitish to dark brown. Specimens reach the final coloration after 5 days from adult emergence.
**Changes in second sternal complex** (Figure 3): the apodemes show an increasing in length; being very short at 0 to 1 day old, and reach their maximum length after about 5 days.
**Biological data.** Immature stages and adults of *Zygina nivea* were found at high densities on the abaxial surface of *Populus alba* and *P. nigra* leaves (Figure 4a). Average insect density was 20 insects/leaf; while 98% of the leaves randomly selected had insects.
  *Z. nivea* feeding damage on poplar leaves was seen as abundant chlorotic areas, which are more evident in adaxial side of the leaf blade (Figure 4b). Insect feeding was also correlated with the excretion of greenish honeydew drops (Figure 4c). Nymphs and adults showed behavior of washing, whereby the insects spread fluids excretion over the tegmina, scutellum, pronotum, and head; using first both hind legs and then the forelegs on the head (Video 1).
**Distribution:** Algeria, Austria, Belgium, Czechoslovakia, France, Germany, Greece, Hungary, Italy, Morocco, Portugal, Spain, Yugoslavia (Dworakowska 1970), Iran (Dlabola 1981), Turkey (Lodos & Kalkandelen 1984), Tunisia, (Linnavuri 1965), Slovenia (Seljak 2004) Moldova, Russia South, Slovakia, Ukraine (Fauna Europaea), and Argentina.
**Host Plants:** *Zygina nivea* has been reported on *P. alba, P. nigra, Salix alba, S. eleagnos, S. incana* (Dworakowska 1970; Linnavuori 1965; Seljak 2004), *Vitex agnus* (Ribaut 1936; Linnavuori 1962; Emelyanov 1964; Dworakowska 1970); *Vitis vinifera* (Lodos & Kalkandelen 1984; Altinçağ & Akten 1993); *Lactuca sativa, Brassica oleracea* (Nebreda 2005). In Argentina, it is found on *P. alba* and *P. nigra.*

**Video 1.**
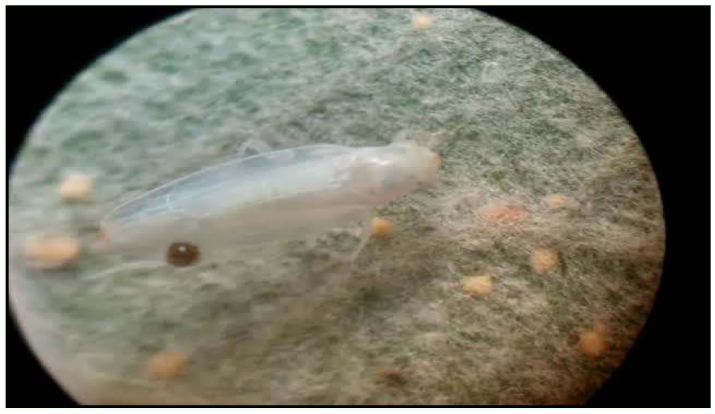
Click image to view video. Download Video

## Discussion

Erythroneurini fauna of the New World comprise more than 700 described species (Dietrich and Dmitriev 2006); however, the Argentinean Erythroneurini is very poorly known. This could be due to a lack of intensive samplings of these species.

The polymorphism of body coloration and shape of the second sternal complex of this species resembles that of *Zygina flammigera* (Fourcroy 1785) as described by Günthart (1979). Many species of this tribe are known to exhibit considerable color polymorphism, and for this reason several taxa previously based on coloration, but lacking distinct differences in the male genitalia, have been subsequently treated as synonyms.

The feeding damage produced by *Zygina nivea* on poplar leaves resembles the characteristic symptom described by Backus et al (2005) for typhlocybine “stippler” species, as *Empoasca abrupta* DeLong and *Zyginidia scutellaris* Herrich-Schaeffer. The damage observed, in addition to the presence of greenish honeydew drops, could be a result of feeding from mesophyll cells contents as in other species (Marion Poll et al. 1987; Backus et al. 2005; Brentassi et al. 2010).

The behaviour of washing shown by *Z*. *nivea* may be a kind of anointing; similar behaviour has been described by Rakitov (1996) for other typhlocybines.

The symptoms of feeding damage to the leaves and the high densities of *Zygina nivea* observed on *P. alba* and *P. nigra* plants suggest that this species has potential pest status because the use of poplar trees for wood production and in public spaces has increased in recent years.
